# The Role of Urinary *N*-Acetyl-β-d-glucosaminidase in Cirrhotic Patients with Acute Kidney Injury: Multicenter, Prospective Cohort Study

**DOI:** 10.3390/jcm10194328

**Published:** 2021-09-23

**Authors:** Jeong-Ju Yoo, Jung Hyun Kwon, Young Seok Kim, Soon Woo Nam, Ji Won Park, Hee Yeon Kim, Chang Wook Kim, Seung Kak Shin, Young Eun Chon, Eun Sun Jang, Sook-Hyang Jeong, Jin Woo Lee, Do Seon Song, Jin Mo Yang, Sung Won Lee, Hae Lim Lee, Young Kul Jung, Hyung Joon Yim, Bora Lee, Sang Gyune Kim, Ju Hyun Kim

**Affiliations:** 1 Department of Gastroenterology and Hepatology, Soonchunhyang University School of Medicine, Seoul 14584, Korea; puby17@schmc.ac.kr (J.-J.Y.); liverkys@schmc.ac.kr (Y.S.K.); 2 Department of Internal Medicine, Incheon St. Mary’s Hospital, The Catholic University of Korea, Incheon 21431, Korea; doctorkwon@catholic.ac.kr (J.H.K.); drswnam@catholic.ac.kr (S.W.N.); 3 Department of Internal Medicine, Hallym University Sacred Heart Hospital, Hallym University College of Medicine, Anyang 14068, Korea; miunorijw@hanmail.net; 4 Department of Internal Medicine, Uijeongbu St. Mary’s Hospital, College of Medicine, The Catholic University of Korea, Seoul 11765, Korea; hee82@catholic.ac.kr (H.Y.K.); cwkim@catholic.ac.kr (C.W.K.); 5 Department of Internal Medicine, Gil Medical Center, Gachon University College of Medicine, Incheon 21565, Korea; puressk@gilhospital.com; 6 Department of Internal Medicine, Cha Bundang Medical Center, Cha University, Seongnam 13496, Korea; nachivysoo@chamc.co.kr; 7 Department of Internal Medicine, Seoul National University Bundang Hospital, Seoul National University College of Medicine, Seongnam 13620, Korea; janges@snubh.org (E.S.J.); jsh@snubh.org (S.-H.J.); 8 Department of Internal Medicine, Inha University School of Medicine, Incheon 22212, Korea; jin@inha.ac.kr; 9 Department of Internal Medicine, St. Vincent’s Hospital, The Catholic University of Korea, Suwon 16247, Korea; dsman@catholic.ac.kr (D.S.S.); jmyangdr@catholic.ac.kr (J.M.Y.); 10 Department of Internal Medicine, Bucheon St. Mary’s Hospital, College of Medicine, The Catholic University of Korea, Seoul 06591, Korea; swleehepa@gmail.com (S.W.L.); leehaelim@catholic.ac.kr (H.L.L.); 11 Department of Internal Medicine, Korea University Ansan Hospital, Ansan 15355, Korea; 93cool@hanmail.net (Y.K.J.); gudwns21@korea.ac.kr (H.J.Y.); 12 Department of Statistics, Chung-Ang University, Seoul 06974, Korea; mintbora0125@gmail.com

**Keywords:** acute kidney injury, hepatorenal syndrome, *N*-acetyl-β-d-Glucosaminidase

## Abstract

Background and Aims: Currently, it is difficult to predict the reversibility of renal function and to discriminate renal parenchymal injury in cirrhotic patients with acute kidney injury (AKI). The aim of this study is to evaluate whether urine *N*-acetyl-β-d-Glucosaminidase (NAG) can predict the survival and response to terlipressin in cirrhotic patients with AKI. Methods: Two hundred sixty-two cirrhotic consecutive patients who developed AKI were prospectively enrolled from 11 tertiary medical centers in Korea between 2016 to 2019. AKI was defined as an increase in serum Cr (SCr) of 0.3 mg/dL or a 50% increase in baseline SCr. Patients diagnosed with hepatorenal syndrome (HRS-AKI) were treated with terlipressin plus albumin. Results: The patients were 58.8 ± 12.9 years old on average and were predominantly male (72.5%). The mean MELD score was 25.3 ± 9.1. When classified according to the AKI phenotype, there were 119 pre-renal, 52 acute tubular necrosis, 18 miscellaneous, and 73 HRS-AKI patients. However, the urine NAG was not effective at discriminating AKI phenotypes, except for HRS-AKI. The baseline urine NAG increased as the baseline AKI stage increased (*p* < 0.001). In addition, within the same AKI stage, the urine NAG values were significantly lower in the AKI-resolved group than in the unresolved group. The urine NAG level was significantly lower in living patients compared with those who died or who underwent a liver transplant within 3 months (*p* = 0.005). In the multivariate analysis, the increased urine NAG was a significant risk factor for the 3-month transplant-free survival (TFS) rate, especially in patients with Child–Pugh class ≤ B or MELD < 24. The urine NAG did not predict the response to terlipressin treatment in patients with HRS. Conclusions: Urine NAG is strongly associated with the severity of AKI in patients with liver cirrhosis and is useful for predicting the 3-month TFS.

## 1. Introduction

Occurring in 40–50% of hospitalized patients, acute kidney injury (AKI) is a common complication in patients with advanced liver cirrhosis [1,2]. Numerous studies have established that kidney function plays a major role in the prognosis of cirrhosis [3]. Firstly, it is related to the increasing development of other complications, including variceal bleeding, spontaneous bacterial peritonitis, and hepatic encephalopathy for about 1.2–1.5 times [4]. Secondly, AKI shortens the survival time of patients with liver cirrhosis and is a common cause of mortality [3]. Referring to previous studies, even stage 1 AKI can be life-threatening in advanced liver cirrhosis. If AKI does not improve during the early stages of cirrhosis, the 3-month mortality rate can reach 32% [5]. Therefore, early identification and treatment may facilitate recovery from AKI, thereby reducing mortality in cirrhosis patients.

Serum creatinine (SCr) is the most widely used tool for the diagnosis of AKI [6]. Although SCr is the simplest tool to use in clinical practice, its accuracy in patients with cirrhosis has been questioned. A recent study showed that renal dysfunction is greatly underestimated on the basis of creatinine levels in cirrhotic patients [7]. As SCr may not be as competent as expected for predicting the prognosis of AKI patients, an alternative is required.

The definition and management of hepatorenal syndrome (HRS-AKI), the most life-threatening form of AKI, have changed as we have gained new knowledge into the pathophysiology [8]. While terlipressin plus albumin is the standard treatment for HRS-AKI, it only works in about half of treated patients. To date, the predictive factors associated with terlipressin response are a high baseline SCr, high bilirubin, and lack of increase in mean arterial pressure during treatment [9]. Given these facts, clinicians of late have focused on finding new biomarkers for the accurate diagnosis of AKI, differentiation of the type of AKI, and the prediction of terlipressin response in HRS-AKI.

The usefulness of biomarkers such as Neutrophil Gelatinase Associated Lipocalin (NAGL), interleukin-18, kidney injury molecule-1, and L-FABP have been studied in patients with cirrhosis [10]. Unfortunately, these biomarkers have non-specific increases under systemic inflammation, urinary tract infection, etc., and the ability to distinguish between HRS-AKI and acute tubular necrosis (ATN) is modest. Urine *N*-acetyl-β-d-glucosaminidase (NAG) is a lysosomal enzyme present dominantly in proximal tubules. It has a high molecular weight (140 kDa) and cannot penetrate to the glomerulus, meaning it is unlikely to be increased due to damage outside the kidney. Urine NAG has been proven to be clinically effective for the prediction of acute renal failure, post cardiac surgery, and in primary glomerulonephritis [11]. Despite this clear clinical relevance, little is known about how well urine NAG predicts the prognosis and treatment response in cirrhotic patients with AKI. The aim of this study was to evaluate the usefulness of the urine biomarker NAG for predicting the survival and response to terlipressin in patients with liver cirrhosis and established AKI or HRS-AKI.

## 2. Materials and Methods

### 2.1. Patients and Study Protocol

This is a multicenter, prospective cohort study involving 11 tertiary referral hospitals during the period of April 2017 to August 2019. The study protocol was registered at ClinicalTrials.gov (registration number NCT03530761, accessed on 13 August 2021). Reporting of the study conforms to the Consolidated Standards of Reporting Trials (CONSORT) 2010 statement. The inclusion criteria were as follows: (1) hospitalized patients with liver cirrhosis who were over 19 years old, (2) accompanied by acute kidney injury or hepatorenal syndrome, (3) able to urinate, and (4) agreed to the study. Exclusion criteria were as follows: (1) patients with active bleeding (e.g., variceal bleeding) within last 7 days from enrollment, (2) prior or current history of hepatocellular carcinoma (HCC), (3) hypersensitivity to terlipressin, (4) chronic kidney disease, (5) acute on chronic liver failure without liver cirrhosis at enrollment, (6) inadequate urine sample for examination, (7) pregnant and lactating patients, or (8) those who did not agree to participate in the study. The study protocol was approved by the Institutional Review Board of each hospital (IRB number; SCHBC 2017-02-005-003). The study protocol conformed to the ethical guidelines of the World Medical Association’s Declaration of Helsinki. All participants of the provided written informed consent. All authors had access to the study data and reviewed and approved the final manuscript. 

### 2.2. Data Collection and Measurement of Urine NAG

In all of the patients, the baseline urine NAG was measured before the administration of albumin. In the case of HRS patients receiving terlipressin, urine NAG levels were rechecked 3 days after the standard treatment of terlipressin plus albumin. Urine NAG was measured using a colorimetric assay with a commercially available kit (N-Assay L NAG Nittobo, Nittobo Medical Co. Ltd., Tokyo, Japan) and automatic blood analyzer (Accute (TBA-40FR), TOSHIBA-CANON, Tochigi, Japan). Other clinical information including baseline demographics, laboratory data, and clinical course were collected prospectively.

### 2.3. Definition, Management, Outcomes, and Follow-Up

The definition of AKI and HRS followed the current definition of ICA-AKI criteria [6]. AKI was defined as an increase in serum creatinine of more than 0.3 mg/dL within 48 h or a 50% increase from baseline. The phenotypes of the AKI were classified into four groups as follows [12,13,14]: (1) pre-renal AKI, history of acute hypovolemia such as diuretics, bleeding, or diarrhea; (2) ATN [8,15,16], fractional excretion of sodium (FeNa) > 2% or urine sodium > 40 mEq/L or urine osmolarity < 350 mOsm/L; (3) HRS-AKI was defined by (i) diagnosis of cirrhosis and ascites, (ii) AKI according to ICA-AKI criteria [6], (iii) no response after two consecutive days of diuretic withdrawal and plasma volume expansion with 1 g/kg albumin, (iv) absence of shock, (v) no current or recent use of nephrotoxic drugs, and (vi) no macroscopic signs of structural kidney injury, absence of microhematuria, and normal findings on renal ultrasonography; and (4) miscellaneous, not included in any of the above three groups.

Management of AKI or HRS followed the standard treatment of the current guidelines [6,17,18], including fluid resuscitation, withdrawal of nephrotoxic drug or diuretics, or volume expansion with albumin.

Primary outcomes were a 3-month transplant-free survival in all AKI patients and response rate to terlipressin therapy in HRS-AKI patients. Secondary outcomes were overall survival, regression of AKI, and recurrence of AKI. The index date was defined as the date when an AKI or HRS occurred. The follow-up period was calculated from the index date to the day when the outcome of interest occurred or the last follow-up date.

### 2.4. Sample Size Calculation

Given the lack of previous research into this topic, we referred to a related study reporting the area-under-the curve (AUC) for urine biomarkers in patients with AKI, as it relates to the overall mortality (AUC for mortality was 0.75) [19]. We conservatively hypothesized that urine NAG would have a similar predictability. The estimated sample size was 220 patients, with an alpha value of 0.05 and a power of 80%. Considering a 10% drop out rate, a total of 245 patients were required. In patients with HRS-AKI, a subgroup of this study, the sample size was calculated based on the response to terlipressin therapy. According to a previous study, predicting the ability to the response rate of terlipressin was reported to have an AUC of 0.85 [20]. The resulting estimated sample size was a subgroup of 50 patients, with an alpha value of 0.05 and power of 80%. Considering a 10% drop out rate, a total of 56 patients with HRS-AKI were required.

### 2.5. Statistical Analysis

Frequencies and percentages were used for the descriptive statistics. Statistical differences between groups were investigated using χ^2^ test and Student’s *t*-test. Patient survival probability was estimated using the Kaplan–Meier method, and differences between the curves were compared using the log-rank test. The main analysis tool used for survival was the Cox proportional hazards model. Multivariate analysis was performed to determine the effect of urine NAG on liver transplantation or death. In order to minimize the influence of confounding factors, unadjusted (Model 1), age, sex (Model 2), MELD score at baseline (Model 3), and c-reactive protein at baseline (Model 4) were serially adjusted in a Cox proportional hazard model. The confounding factor was selected as items that had a significant effect on liver transplantation or death in univariable analysis (*p* < 0.10) and clinically relevant. In addition, stratification analysis according to the MELD score, Child–Pugh class, and degree ascites were presented. All of the statistical analyses were performed using R (version 4.0.2, The R Foundation for Statistical Computing, Vienna, Austria), SPSS software (ver. 23.0; SPSS Inc., Chicago, IL, USA). Statistical significance was defined at *p* < 0.05.

## 3. Results

### 3.1. Baseline Characteristics and AKI Phenotype

The baseline demographic and clinical characteristics are summarized in Table 1. A total of 262 patients (all hospitalized) were analyzed, including 190 (72.5%) males. The patients were 58.8 ± 12.9 years old on average and had a mean body mass index (BMI) of 24.1 ± 4.2 kg/m^2^. The etiology of liver cirrhosis consisted mainly of alcohol (83.2%) and viral (16.8%). The median follow-up duration was 241 (IQR 40–388) days. Most patients enrolled in the study had deteriorated liver function. The mean Child–Pugh score was 9.8 ± 2.5 and the mean MELD score was 25.3 ± 9.1. AKI was in stage 1 for 135 patients (51.5%) (stage 1A 2.3%, stage 1B 49.2%), stage 2 for 79 patients (30.2%), and stage 3 for 48 patients (18.3%); among these, 73 (27.9%) were HRS-AKI.

When classified according to the AKI phenotype, there were 119 pre-renal, 52 ATN, 18 miscellaneous, and 73 HRS-AKI patients. A comparison of the baseline characteristics according to the AKI phenotype is presented in Table 1. The urine NAG level was higher in the HRS-AKI group than in the other three groups (43.92 ± 65.52 mg/dL, *p* = 0.002). In the remaining three groups, except for HRS-AKI, the urine NAG level was 26.36 ± 37.06 mg/dL in the pre-renal group, 16.06 ± 23.40 mg/dL in the ATN group, and 13.22 ± 16.52 mg/dL in the miscellaneous group, showing no significant difference between groups (*p* = 0.076). Differences in transplant free survival (TFS) according to AKI phenotypes were compared, but no differences were found between the groups (log ran *p* = 0.477) (Supplementary Figure S1).

### 3.2. Relationship between Urine NAG and AKI Stage

**Figure 1:**
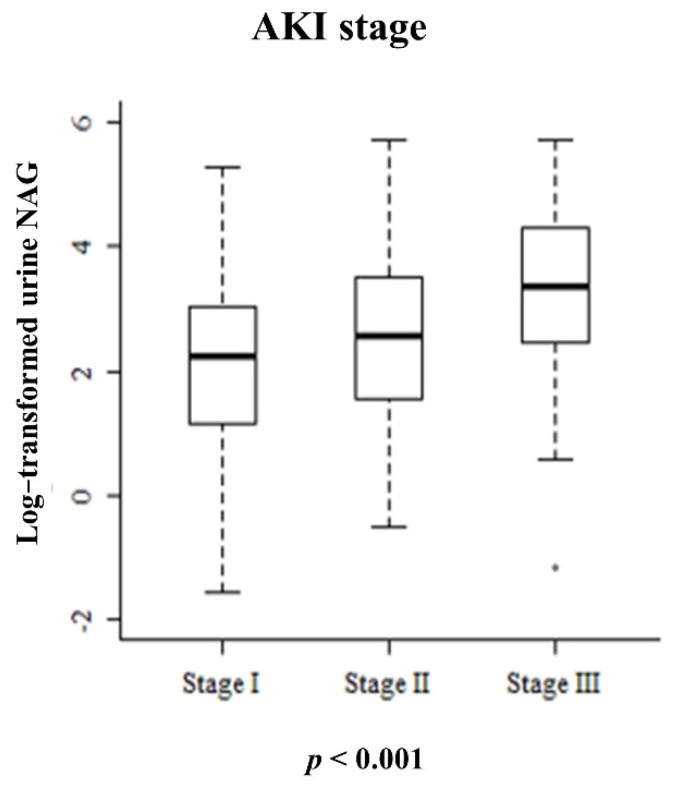
Boxplot comparing the mean urine NAG according to AKI stage.

The mean urine NAG value was 28.31 ± 45.23 mg/dL. Urine NAG increased as the baseline AKI stage increased. The mean urine NAG values according to each AKI stage were 17.22 ± 24.66, 32.11 ± 52.71, and 53.23 ± 63.27 mg/dL, respectively, which were significantly different (Figure 1, *p* < 0.01). The baseline values of the urine NAG were compared between 87 patients with diabetes and the remaining patients without diabetes, but there was no clinically significant difference.

### 3.3. Relationship of Urine NAG and Change of AKI Status

**Figure 2:**
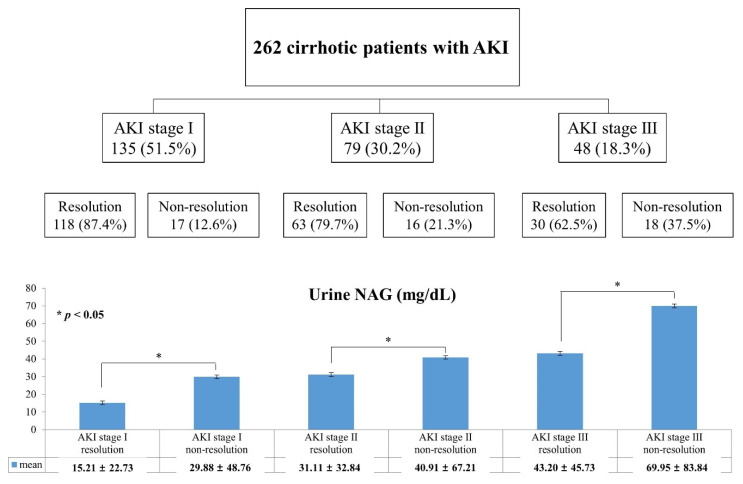
Flowchart of AKI status and urine NAG according to AKI regression. (* *p* < 0.05).

Figure 2 depicts the change of AKI status during follow-up. The patients with regressed AKI were 118 (87.4%) at stage 1, 63 (79.7%) at stage 2, and 30 (62.5%) at stage 3. As the AKI stage increased, the proportion who showed regression significantly decreased (*p* < 0.01). Notably, the urine NAG values were significantly lower in the resolved group than in the unresolved group within each AKI stage (AKI stage I, 15.21 ± 22.73 vs. 29.88 ± 48.76 mg/dL, *p* = 0.027; AKI stage II, 31.11 ± 32.84 vs. 40.91 ± 67.21 mg/dL, *p* = 0.043; and AKI stage III, 43.20 ± 45.73 vs. 69.95 ± 83.84 mg/dL, *p* = 0.032). This result suggests that urine NAG is useful for predicting who can recover from AKI.

During the follow-up period, 93 (35.5%) patients had AKI recurrence. However, no difference in urine NAG was observed according to the recurrence of AKI (Supplementary Figure S2, *p* = 0.180). The logistic regression analysis also showed that the urine NAG level was not associated with the recurrence of AKI (Supplementary Table S1).

### 3.4. Relationship between Urine NAG and 3-Months Transplant-Free Survival

**Figure 3:**
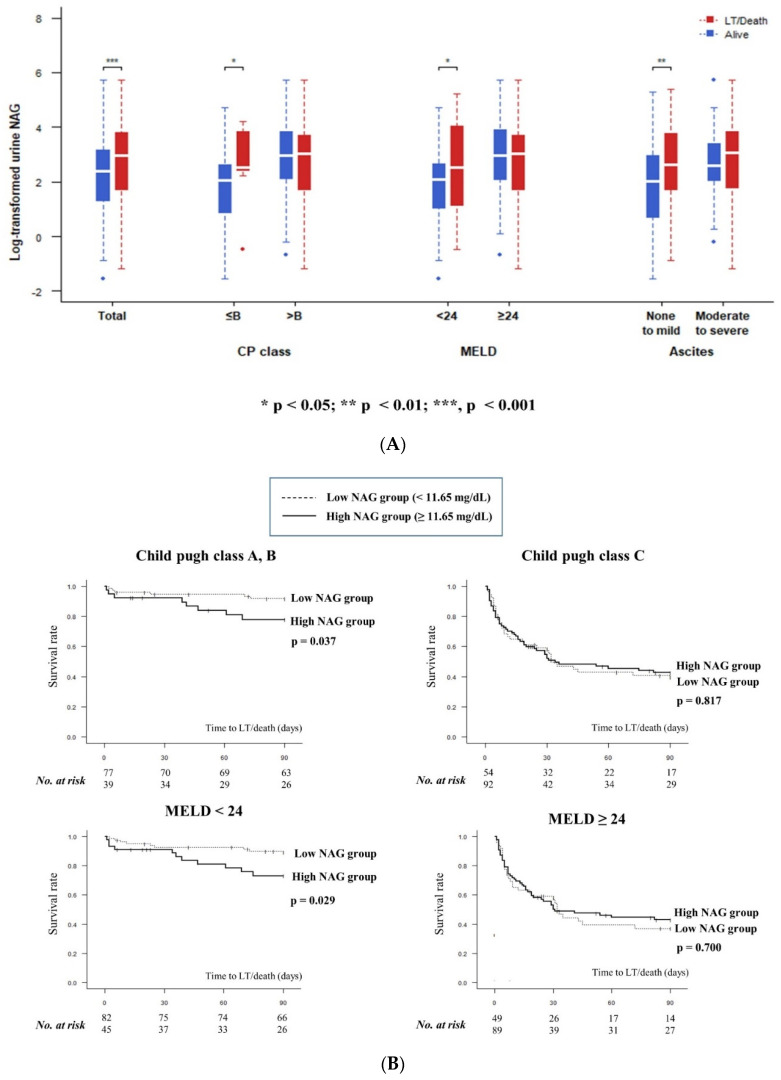
Urine NAG and liver disease severity: (**A**) comparison of urine NAG values; (**B**) transplant-free survival according to urine NAG.

During the 3-month follow-up period, 95 patients died or received liver transplants. The baseline characteristic comparison of the two groups is presented in Table 2. Patients who experienced death or liver transplant within 3 months showed significantly higher urine NAG than those who did not (38.80 ± 55.90 vs. 22.34 ± 36.73 mg/dL, *p* = 0.024, Supplementary Figure S3). The multivariate analysis also showed that urine NAG was a significant prognostic marker of death or transplantation within 3 months (Table 3). Of note, urine NAG showed more predictive power in the patients with preserved liver function. The performance of urine NAG for predicting the 3-month TFS was slightly different according to the severity of liver disease. Among the patients with a relatively good liver function, those who were alive had lower urine NAG values compared with those who were not. In patients with deteriorated liver function, such as those with Child–Pugh class C, MELD score over 24, or those with significant ascites, no significant difference in urine NAG values according to clinical outcomes were found (Figure 3A, Supplementary Table S2). Looking at the Kaplan–Meier curve, high urine NAG is related to an increased risk of poor transplant-free survival in patients with Child–Pugh class A or B, or with a MELD below 24 (Figure 3B).

### 3.5. Urine NAG and Terlipressin Treatment Response in HRS-AKI

Of the total AKI patients, 73 patients (27.86%) were classified as HRS-AKI. The baseline characteristics of these patients are presented in Table 1. There was no significant relationship between the clinical outcome and urine NAG value in HRS-AKI patients. There was no difference in urine NAG according to AKI regression (*p* = 0.663), AKI recurrence (*p* = 0.216), 3-month TFS (*p* = 0.689), and overall survival (*p* = 0.868) (Supplementary Figure S4).

One of our main interests in HRS-AKI patients was whether this biomarker could predict the response to terlipressin treatment. Urine NAG tended to decrease on day 3 in the terlipressin-response group, but it was not statistically significant (*p* = 0.383, Supplementary Figure S5). The logistic regression analysis also showed that the urine NAG level was not effective at predicting the response to terlipressin in patients with HRS-AKI (Supplementary Table S3).

## 4. Discussion

This multicenter, prospective study included more than 200 patients and showed that urine NAG is associated with baseline AKI stage and the short-term prognosis of cirrhotic patients with AKI. Our results indicate that in patients with established AKI, the urinary NAG activity is a useful surrogate for the severity of AKI and that it has prognostic utility.

AKI is still often missed in patients with liver cirrhosis, while the pathophysiology is still being actively studied. Since the ICA criteria for patients with liver cirrhosis was first introduced in 2005, the definitions and pathophysiology of AKI and HRS have continued to evolve as the version was updated in 2015 and 2019 [6,8,21]. It has been found that the increase of SCr is more important than the absolute value of SCr, and that inflammation plays an important role in AKI/HRS pathophysiology outside of the traditional hemodynamic changes [6,8]. As the research progresses, biomarker studies for detecting AKI early and differentiating between prerenal azotemia and ATN are being actively conducted.

Currently, the most representative biomarkers are NGAL and IL-18. Previous reports have shown that the presence of ATN significantly increases the level of these renal biomarkers compared with the prerenal azotemia [22,23]. However, NGAL and IL-18 have several disadvantages as they increase during non-AKI situations and are insufficient to differentiate HRS from ATN [10,24]. Ideal prerequisites for any clinically useful AKI marker include the following: (1) the biomarker should only be found in the AKI state, (2) the degree of elevation is related to the severity, (3) the value should be higher for ATN than for either PRA or HRS, and (4) it should be accessible in the clinic and testing costs should be as low as creatinine testing.

In this context, NAG could be a good candidate as a biomarker for AKI. NAG is a large molecule (130 kDa) and cannot penetrate the glomerulus [11,25]. Therefore, it is only elevated during renal tubular injury, suggesting that it is more specific for discriminating ATN. On top of this, the concentrations of NAG were significantly higher in patients with AKI compared with the controls or with chronic kidney disease [26,27]. Thanks to its proven usefulness for post cardiac surgery and more generally for AKI patients without cirrhosis, it is now commercially available and relatively inexpensive compared with other biomarkers. Testing costs are as low as $10 [11,28].

There is only a one retrospective study on the usefulness of NAG in patients with cirrhosis, which was conducted in decompensated cirrhotic patients [29]. However, the number of patients was relatively small and the proportion of patients with AKI was too low, so it was insufficient to confirm the usefulness of NAG in AKI patients. As far as we know, our study is the first prospective study in patients with liver cirrhosis who have already developed AKI or HRS.

The first finding in our study was that urine NAG was associated with short term survival in AKI patients. This was clinically significant, even after correcting for other factors such as liver function. This means that the higher the NAG level, the higher the mortality rate. This is similar to other studies, where a high NAG has been reported to be associated with dialysis or death in ARF patients without liver cirrhosis [11]. Although NGAL and IL-18 have demonstrated their usefulness in predicting the prognosis of cirrhosis patients and in patients with acute-on-chronic liver failure, both markers are not widely used in clinical practice [22]. It is difficult for hepatologists to predict which patients will recover from AKI and which will not. Therefore, for AKI patients requiring rapid diagnosis and treatment, NAG is clearly more predictive than Cr. In other words, our results suggest that patients with increased urine NAG are likely to have a poor prognosis and will not to respond to treatment, making timely liver transplant a priority.

The second finding of our study is that the information provided by urine NAG is not very useful in patients with highly advanced decompensated cirrhosis. This makes sense as the liver function itself is a more important factor for determining prognosis than renal function in patients with advanced decompensated liver cirrhosis. For this reason, Cr has a lower priority than the previous items [30]. The most commonly used predictors reported in the literature are in the order of bilirubin, prothrombin time, albumin, ascites, and age, which is the same as for our study [30]. In addition, urine NAG is known to reflect mild, subclinical renal tubular damage better than severe tubular damage [31,32]. Therefore, it is sensible it showed high predictive power during the early stages of liver cirrhosis. Thus, we would like to recommend liver transplantation or best supportive care for these patients with severe grade (Child–Pugh class C or with high MELD score) liver cirrhosis [1,33].

Finally, our study found that there was no predictive power of urine NAG in patients with HRS-AKI receiving terlipressin. Similarly, urine NGAL failed to predict the response to terlipressin treatment in patients with HRS [24]. We presume that there are three reasons NAG failed to predict the terlipressin response. In HRS-AKI, systemic inflammation and systemic circulatory dysfunction was found to be important for pathophysiology [8]. Because the urine NAG level is generally not associated with systemic inflammation, it appears that the predictive power of NAG is diminished in HRS-AKI patients. As a second hypothesis, it is possible that a significant number of patients diagnosed with HRS-AKI actually overlapped with ATN, as HRS and ATN are on a continuous spectrum [6]. In these heterogeneous groups, prediction of the response to treatment may vary depending on the timing and degree of ATN manifestation. Finally, as the treatment response was not the primary aim of this study, there is a possibility of beta error due to an insufficient sample size, and the patients events occurred outside the hospital, so there may have been a difference in the sampling time. Perhaps we more meaningful results would be produced if serial sampling is possible in hospitalized patients. To date, clinical factors such as serum bilirubin, early increase in arterial pressure, and the development of hypotension have been reported as predictors of the response to terlipressin [34,35], but favorable results have not been reported in the biomarker field.

As with all studies, there are limitations to our research. First, contrary to the results of other biomarkers, urine NAG was not effective at discriminating AKI phenotypes, except for HRS-AKI. In previous studies, the AKI phenotype was reported to be pre-renal 48%, ATN 12%, and HRS-AKI 29% [24], and it is almost similar to the classification in our study (pre-renal 45.4%, ATN 19.8%, and HRS-AKI 28%). As renal biopsy was not done in the patients with suspected ATN, we tried to discriminate it indirectly through FeNa, urine sediment, urine osmolality, and urine sodium. While these are commonly used metrics, it is well known that these clinical indicators are less accurate in cirrhotic patients [36,37,38,39]. Moreover, there might be significant overlap between HRS-AKI and ATN, and classifying the exact type of AKI may be clinically inaccurate in this heterogeneous group. As a result, we think that this inaccurate classification may have lowered the phenotype predictive power of urine NAG. Second, we measured the urine NAG at baseline and on day 3 after treatment in order to predict the response to terlipressin. In fact, when the measurement should be performed for the highest accuracy has yet to be validated. In other research, measurement on the second day after the diagnosis of HRS is recommended, but more research is needed in the future [40].

Clinically, we expect urine NAG to be used as a diagnostic tool to predict the prognosis of patients with AKI and HRS. Urine NAG can help identify patients who need to prioritize a liver transplant rather than pharmacological treatment.

In conclusion, this is the first study to demonstrate the clinical utility of urine NAG as a short-term prognostic marker in cirrhotic patients with AKI or HRS-AKI. A more precise estimation of the severity of AKI and the ability to predict clinical outcomes using urine NAG can potentially facilitate targeted therapies for patients who have advanced liver cirrhosis.

## Figures and Tables

**Table 1:** Baseline characteristics and AKI phenotype.

Variable	Total (*N* = 262)	Pre-Renal (*N* = 119)	ATN (*N* = 52)	Miscellaneous (*N* = 18)	HRS-AKI (*N* = 73)	*p*
Age (year)	58.8 ± 12.9	59.0 ± 13.1	60.6 ± 13.7	62.6 ± 13.0	56.1 ± 11.6	0.121
Male (*n*, %)	190 (72.5%)	96 (80.7%)	38 (73.1%)	10 (55.6%)	46 (63.0%)	0.020
Body mass index (kg/m^2^)	24.0 ± 4.2	24.0 ± 4.1	23.7 ± 4.6	24.0 ± 4.3	24.2 ± 4.1	0.931
Etiology (*n*, %)						0.418
Viral	44 (16.8%)	15 (12.6%)	10 (19.2%)	4 (22.2%)	15 (20.5%)	
Non-viral	218 (83.2%)	104 (87.4%)	42 (80.8%)	14 (77.8%)	58 (79.5%)	
Current alcohol drinking (*n*, %)	100 (38.1%)	42 (35.3%)	14 (26.9%)	6 (33.3%)	25 (34.2%)	0.209
Diabetes (*n*, %)	87 (33.2%)	38 (31.9%)	21 (40.4%)	4 (22.2%)	24 (32.9%)	0.517
Prior use of diuretics (*n*, %)	152 (58.0%)	71 (59.7%)	27 (51.9%)	13 (72.2%)	41 (56.2%)	0.472
Prior use of beta blocker (*n*, %)	55 (20.9%)	27 (22.7%)	12 (23.1%)	3 (16.7%)	13 (17.8%)	0.803
AKI stage (*n*, %)						<0.001
Stage I	135 (51.5%)	75 (63.0%)	25 (48.1%)	13 (72.2%)	NA	
Stage II	79 (30.1%)	30 (25.2%)	19 (36.5%)	4 (22.2%)	NA	
Stage III	48 (18.4%)	14 (11.8%)	8 (15.4%)	1 (5.6%)	NA	
HRS-AKI (*n*, %)	73 (27.8%)	0 (0%)	0 (0%)	0 (0%)	73 (100%)	<0.001
Ascites (*n*, %)						0.081
No	84 (32.1%)	36 (30.3%)	25 (48.1%)	6 (33.3%)	17 (23.3%)	
Mild	47 (17.9%)	21 (17.6%)	10 (19.2%)	4 (22.2%)	12 (16.4%)	
Moderate to severe	131 (50.0%)	62 (52.1%)	17 (32.7%)	8 (44.4%)	44 (60.3%)	
Hepatic encephalopathy (*n*, %)						0.125
No	199 (76.0%)	88 (73.9%)	46 (88.5%)	16 (88.9%)	49 (67.1%)	
Grade I to II	26 (9.9%)	14 (11.8%)	2 (3.8%)	1 (5.6%)	9 (12.3%)	
Grade III to IV	37 (14.1%)	17 (14.3%)	4 (7.7%)	1 (5.6%)	15 (20.5%)	
Child–Pugh class (*n*, %)						0.002
A	28 (10.7%)	10 (8.4%)	11 (21.2%)	5 (27.8%)	2 (2.7%)	
B	88 (33.6%)	43 (36.1%)	20 (38.5%)	3 (16.7%)	22 (30.1%)	
C	146 (55.7%)	66 (55.5%)	21 (40.4%)	10 (55.6%)	49 (67.1%)	
Child–Pugh score	9.8 ± 2.4	9.9 ± 2.3	8.6 ± 2.5	8.8 ± 2.6	10.6 ± 2.2	<0.001
MELD score	25.2 ± 9.1	24.1 ± 7.9	22.4 ± 8.6	21.1 ± 9.1	30.2 ± 9.3	<0.001
Vital sign						
Systolic blood pressure (mmHg)	116 ± 19	114 ± 18	120 ± 20	119 ± 17	114 ± 19	0.255
Diastolic blood pressure (mmHg)	69 ± 14	69 ± 12	70 ± 11	74 ± 12	69 ± 17	0.486
Mean blood pressure (mmHg)	85 ± 14	84 ± 13	87 ± 14	89 ± 13	84 ± 17	0.435
Heart rate (beats per minute)	86 ± 17	87 ± 18	83 ± 17	86 ± 17	85 ± 16	0.517
Laboratory findings						
White blood cell (/μL)	9748 ± 6898	10,727 ± 7691	8589 ± 6606	6173 ± 3228	9860 ± 3562	0.032
Hemoglobin (g/dL)	9.7 ± 2.2	9.7 ± 2.3	9.9 ± 2.6	10.2 ± 1.4	9.5 ± 2.1	0.542
Platelet (10^3^/μL)	104 ± 60	105 ± 63	113 ± 61	104 ± 64	98 ± 55	0.602
hs-CRP (mg/dL)	3.2 ± 4.3	3.5 ± 3.8	2.8 ± 4.9	2.4 ± 4.2	3.1 ± 4.5	0.676
Albumin (g/dL)	2.7 ± 0.6	2.6 ± 0.6	3.0 ± 0.7	2.8 ± 0.8	2.5 ± 0.4	0.002
BUN (mg/dL)	42 ± 23	40 ± 22	35 ± 17	31 ±15	55 ±25	<0.001
Total bilirubin (mg/dL)	8.4 ± 10.1	8.2 ± 10.1	6.6 ± 9.0	3.9 ± 5.0	11.1 ± 11.3	0.016
AST (U/L)	162 ± 621	136 ± 192	296 ± 1320	169 ± 474	107 ± 177	0.363
ALT (U/L)	84 ± 349	64 ± 117	149 ± 708	64 ± 120	75 ± 240	0.510
Serum sodium (mmol/L)	132 ± 7	132 ± 8	133 ± 5	133 ± 6	130 ± 6	0.112
Creatinine_baseline (mg/dL)	1.05 ± 0.36	1.01 ± 0.26	0.92 ± 0.22	1.06 ± 0.29	1.19 ± 0.51	<0.001
Creatinine_enrollment (mg/dL)	2.27 ± 0.87	1.99 ± 0.52	2.01 ± 0.58	1.89 ± 0.76	3.02 ±1.07	<0.001
Prothrombin time (INR)	1.85 ± 0.85	1.78 ± 0.69	1.71 ± 0.75	1.78 ±1.20	2.06 ± 0.99	0.078
Urine NAG (mg/dL)	28.31 ± 45.23	26.36 ± 37.06	16.06 ± 23.40	13.22 ± 16.52	43.92 ± 65.52	0.002

**Table 2:** Comparison of baseline characteristics according to clinical outcomes.

Variable	LT/Death in 3-Months (*N* = 95)	Alive (*N* = 167)	*p*
Age (year)	60.32 ± 12.66	57.96 ± 13.04	0.157
Male (*n*, %)	71 (74.74%)	119 (71.26%)	0.644
Liver transplantation (*n*, %)	13 (13.68%)	0	0.999
Death (*n*, %)	82 (86.31%)	0	0.999
AKI stage (*n*, %)			0.001
Stage I	35 (36.84%)	100 (59.88%)	
Stage II	36 (37.89%)	43 (25.75%)	
Stage III	24 (25.26%)	24 (14.37%)	
HRS-AKI (*n*, %)	36 (37.89%)	37 (22.16%)	0.010
Ascites (*n*, %)			0.021
No	22 (23.16%)	62 (37.13%)	
Mild	15 (15.79%)	32 (19.16%)	
Moderate to severe	58 (61.05%)	73 (43.71%)	
Hepatic encephalopathy (*n*, %)			<0.001
No	59 (62.11%)	140 (83.83%)	
Grade I to II	15 (15.79%)	11 (6.59%)	
Grade III to IV	21 (22.11%)	16 (9.58%)	
Child–Pugh class (*n*, %)			<0.001
A	3 (3.16%)	25 (14.97%)	
B	11 (11.58%)	77 (46.11%)	
C	81 (85.26%)	65 (38.92%)	
Child–Pugh score	11.37 ± 2.11	8.90 ± 2.23	<0.001
MELD score	31.30 ± 8.48	21.84 ± 7.56	<0.001
Laboratory findings			
White blood cell (/μL)	12,270.20 ± 7648.55	8314.59 ± 5999.27	<0.001
Platelet (10^3^/μL)	96.21 ± 58.20	109.65 ± 61.75	0.047
hs-CRP (mg/dL)	4.08 ± 4.52	2.73 ± 4.13	<0.001
Albumin (g/dL)	2.52 ± 0.54	2.86 ± 0.69	<0.001
BUN (mg/dL)	51.18 ± 25.12	38.21 ± 21.38	<0.001
Total bilirubin (mg/dL)	13.25 ± 11.34	5.70 ± 8.31	<0.001
AST (U/L)	159.09 ± 273.73	164.76 ± 750.93	<0.001
ALT (U/L)	89.84 ± 234.14	81.07 ± 401.41	0.003
Serum sodium (mmol/L)	129.79 ± 8.25	133.51 ± 6.21	<0.001
Creatinine_baseline (mg/dL)	1.03 ± 0.37	1.06 ± 0.36	0.392
Creatinine_enrollment (mg/dL)	2.46 ± 0.87	2.17 ± 0.86	0.010
Prothrombin time (INR)	2.37 ± 1.03	1.55 ± 0.52	<0.001
Urine NAG (mg/dL)	38.80 ± 55.90	22.34 ± 36.73	0.005

**Table 3:** Multivariate analysis of the effect of urine NAG on the incidence of LT/death.

Category	*N*	Model 1	Model 2	Model 3	Model 4
		OR (95% CI)	OR (95% CI)	OR (95% CI)	OR (95% CI)
**LT/Death in 3-months**					
Total	262	1.008 (1.002–1.015) **	1.010 (1.004–1.017) **	1.003 (0.997–1.010)	1.003 (0.996–1.010)
MELD					
<24	127	1.028 (1.010–1.052) **	1.039 (1.016–1.065) **	1.036 (1.011–1.063) **	1.034 (1.008–1.062) *
≥24	135	1.000 (0.994–1.007)	1.002 (0.996–1.009)	1.000 (0.993–1.007)	1.000 (0.993–1.007)
Child–Pugh class					
≤B	116	1.022 (1.001–1.044) *	1.033 (1.009–1.059) **	1.029 (1.003–1.055) *	1.028 (1.002–1.054) *
>B	146	1.002 (0.996–1.008)	1.004 (0.997–1.011)	1.001 (0.994–1.008)	1.001 (0.994–1.008)
Ascites					
None to mild	131	1.008 (0.999–1.018)	1.009 (0.999–1.019)	1.000 (0.988–1.012)	1.000 (0.988–1.012)
Moderate to severe	131	1.007 (1.000–1.017)	1.009 (1.001–1.020) *	1.004 (0.995–1.016)	1.003 (0.994–1.013)

* *p* < 0.05; ** *p* < 0.01. Model 1: unadjusted. Model 2: adjusted for age and sex. Model 3: Model 2 plus MELD score at baseline. Model 4: Model 3 plus CRP at baseline.

## Data Availability

The data that support the findings of this study are available upon request from the corresponding author.

## Supplementary Materials

The following are available online at https://www.mdpi.com/article/10.3390/jcm10194328/s1. Table S1: Logistic regression analysis for AKI recurrence in patients with AKI. Table S2: Urine NAG depending upon liver disease severity. Table S3: Baseline characteristics of HRS-AKI. Table S4: Logistic regression analysis for response to terlipressin therapy in patients with HRS-AKI. Figure S1: Transplant free survival according to AKI phenotype. Figure S2: Boxplot comparing mean urine NAG according to AKI recurrence. Figure S3: Urine NAG level according to clinical outcomes. Figure S4: Boxplot comparing mean urine NAG between groups (HRS group). Figure S5: Relationship between delta urine NAG and response to terlipressin.
