# Supplementary material for: The Role of Urinary N-Acetyl-β-d-glucosaminidase in Cirrhotic Patients with Acute Kidney Injury: Multicenter, Prospective Cohort Study
Source: J Clin Med. 2021 Sep 23;10(19):4328. doi: 10.3390/jcm10194328 (PMC8509706; doi:10.3390/jcm10194328)
Supplement: Supplementary file 1 [file jcm-10-04328-s001.zip › jcm-1359997-supplementary.pdf]

Supplementary Material

The role of Urinary Biomarkers in Cirrhotic Patients with Acute Kidney Injury: Multicenter, Prospective Cohort Study

Jeong-Ju Yoo, Jung Hyun Kwon, Young Seok Kim, Soon Woo Nam, Ji Won Park, Hee Yeon Kim, Chang Wook Kim, Seung Kak Shin, Young Eun Chon, Eun-Sun Jang, Sook-Hyang Jeong, Jin Woo Lee, Do Seon Song, Jin Mo Yang, Sung Won Lee, Hae Lim Lee, Young Kul Jung, Hyung Joon Yim, Sang Gyune Kim, Ju Hyun Kim

Table of Contents

I. Supplementary Tables……………………………………………….…………..…13

II. Supplementary Figures....……………..……………………………….…………..….17

I. Supplementary Tables

Supplementary Table S1. Logistic regression analysis for AKI recurrence in patients with AKI

Variable	Univariable		Multivariable	
	HR (95% CI)	p-value	HR (95% CI)	p-value
Age (year)	0.999 (0.984-1.014)	0.896	1.004 (0.985-1.023)	0.699
Male (vs. female)	1.29 (0.821-2.026)	0.269		
BMI (kg/m²)	1.027 (0.982-1.074)	0.25		
Viral etiology (vs. non-viral)	1.786 (1.015-3.143)	**0.044**		
Diabetes	1.275 (0.832-1.954)	0.265		
Prior use of diuretics	0.971 (0.631-1.496)	0.895		
Prior use of beta blocker	0.93 (0.588-1.471)	0.756		
Ascites	0.932 (0.592-1.467)	0.76		
Hepatic encephalopathy	1.871 (1.105-3.169)	**0.02**		
Child-pugh score	1.082 (0.985-1.188)	0.101		
Child-pugh class B-C vs. class A	1.713 (0.883-3.322)	0.111		
MELD score	1.011 (0.987-1.035)	0.375	1.025 (0.993-1.058)	0.130
Systolic blood pressure	0.997 (0.986-1.008)	0.554		
Diastolic blood pressure	0.996 (0.98-1.012)	0.626		
Mean blood pressure	0.996 (0.981-1.011)	0.574		
Heart rate	1.001 (0.992-1.011)	0.786		
WBC	1 (1-1)	0.328		
Hemoglobin	0.977 (0.896-1.066)	0.604		
Platelet	0.999 (0.996-1.003)	0.771		
Hs-CRP	1.07 (1.018-1.124)	**0.007**		
Albumin	0.884 (0.648-1.207)	0.439		
BUN	1.01 (1-1.02)	**0.04**		
Total bilirubin	0.988 (0.964-1.011)	0.3		
AST	1.001 (1-1.002)	0.201		
ALT	1.004 (1.002-1.006)	**0.001**		
Serum sodium	0.96 (0.927-0.995)	**0.025**		
Creatinine	0.911 (0.503-1.652)	0.759		
Prothrombin time (INR)	1.278 (0.938-1.741)	0.12		
Urine NAG	0.995 (0.987-1.003)	0.223	0.993 (0.984-1.001)	0.102

Supplementary Table S2. Urine NAG depending upon liver disease severity

Category	Variable	In 90 days		
		LT/Death	Alive	p-value
Total	N	95	167	
	urine NAG	14.19 (9.79-20.56)	8.08 (6.08-10.76)	**0.005**
MELD < 24	N	20	107	
	urine NAG	8.8 (2.51-30.86)	5.5 (3.92-7.72)	**0.044**
MELD ≥ 24	N	75	60	
	urine NAG	16.11 (11.38-22.82)	16.06 (9.92-26.01)	0.69
Ascites : none to mild	N	37	94	
	urine NAG	14.05 (8.69-22.74)	5.68 (3.76-8.58)	**0.012**
Ascites : moderate to severe	N	58	73	
	urine NAG	14.27 (8.35-24.39)	12.74 (8.86-18.33)	0.253
Child-pugh class : A and B	N	14	102	
	urine NAG	9.06 (1.7-48.39)	5.42 (3.79-7.76)	**0.018**
Child-pugh class : C	N	81	65	
	urine NAG	15.33 (10.82-21.72)	15.14 (9.76-23.48)	0.866

Supplementary Table S3. Logistic regression analysis for response to terlipressin therapy in patients with HRS-AKI

Variable	Univariable		Multivariable	
	OR (95% CI)	p-value	OR (95% CI)	p-value
Age (year)	0.982 (0.942-1.022)	0.377	0.983 (0.941-1.025)	0.414
Male	0.615 (0.233-1.596)	0.32		
BMI (kg/m²)	1.052 (0.939-1.183)	0.384		
Viral	0.467 (0.132-1.485)	0.21		
DM	0.309 (0.103-0.853)	**0.028**		
Use of Diuretic	1.35 (0.534-3.455)	0.527		
Use of Beta blocker	1.956 (0.584-7.128)	0.284		
Ascites	0.565 (0.181-1.68)	0.308		
Hepatic encephalopathy	1.45 (0.545-3.924)	0.457		
SBP	0.989 (0.964-1.012)	0.349		
DBP	0.999 (0.973-1.025)	0.928		
mean BP	0.994 (0.967-1.021)	0.68		
Heart rate	0.983 (0.953-1.011)	0.242		
Urine NAG	0.998 (0.99-1.005)	0.63	0.996 (0.988-1.004)	0.330
WBC	1 (1-1)	0.637		
Hemoglobin	1.111 (0.892-1.402)	0.353		
Platelet	0.999 (0.99-1.007)	0.801		
Hs-CRP	1.102 (0.986-1.271)	0.122		
Albumin	0.871 (0.304-2.462)	0.794		
BUN	1.004 (0.986-1.023)	0.646		
Total bilirubin	1.017 (0.976-1.06)	0.431		
AST	1.001 (0.998-1.004)	0.637		
ALT	0.998 (0.992-1.001)	0.445		
serum-Na	0.95 (0.882-1.018)	0.157		
Creatinine	0.098 (0.017-0.377)	**0.003**		
PT (INR)	1.064 (0.664-1.72)	0.791		
CP score	1.164 (0.945-1.453)	0.162		
CP class (B-C)	15216600.842 (0-NA)	0.992		
MELD score	1.042 (0.991-1.1)	0.116	1.045 (0.992-1.106)	0.106

II. Supplementary Figures

Supplementary Figure S1. Transplant free survival according to AKI phenotype

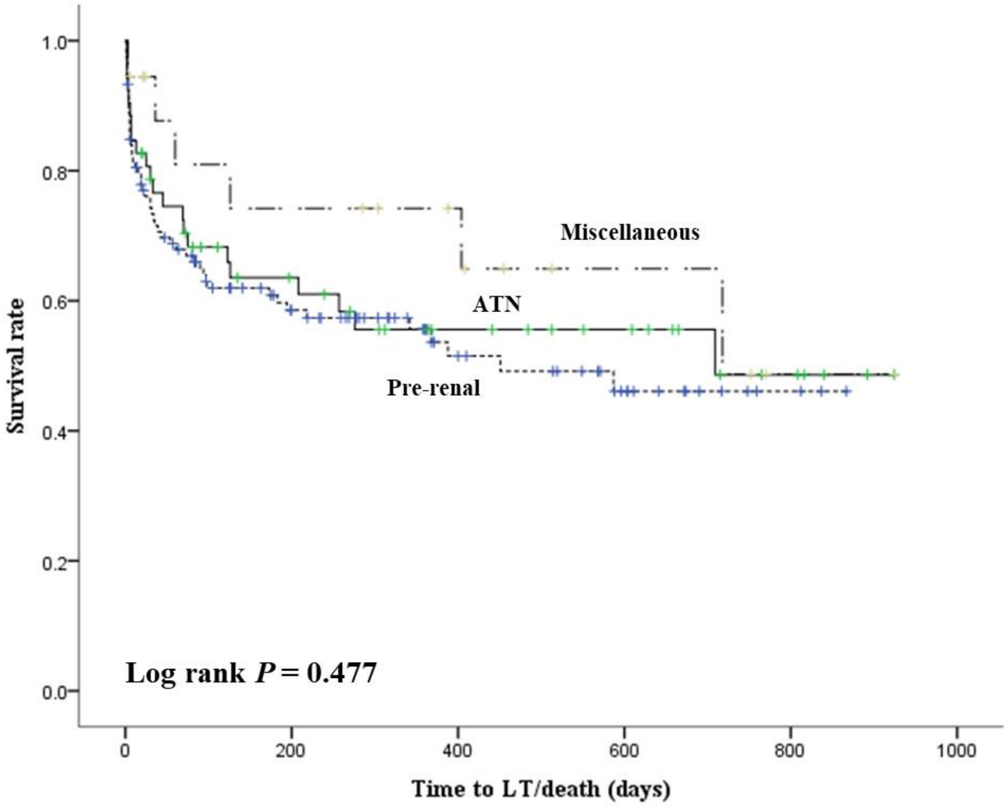

Supplementary Figure S2. Boxplot comparing mean urine NAG according to AKI recurrence

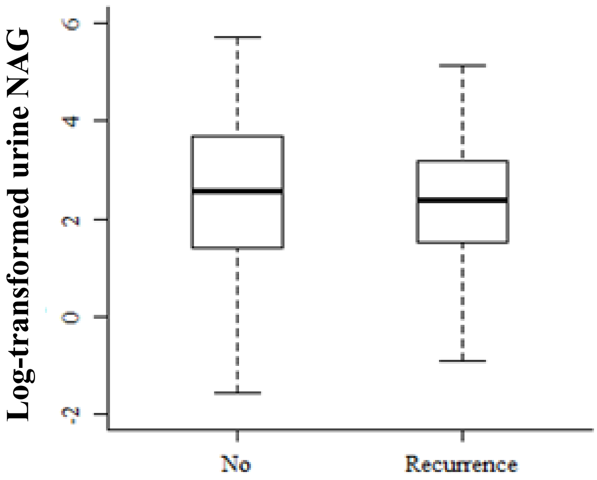

P **= 0.180**

Supplementary Figure S3. Urine NAG level according to clinical outcomes

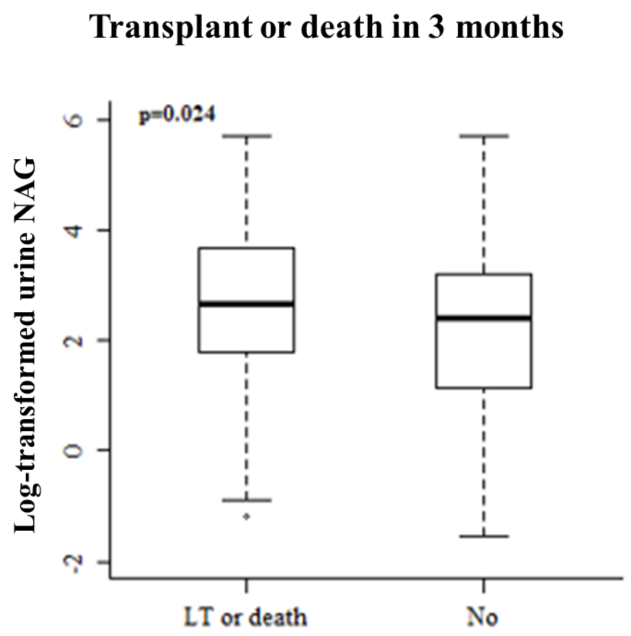

**Urine NAG (mg/dL)**	**38.80 ± 55.90**	**22.34 ± 36.73**	***P* = 0.024**

Supplementary Figure S4. Boxplot comparing mean urine NAG between groups (HRS group)

AKI Improvement

AKI Recurrence

Survival

LT or death

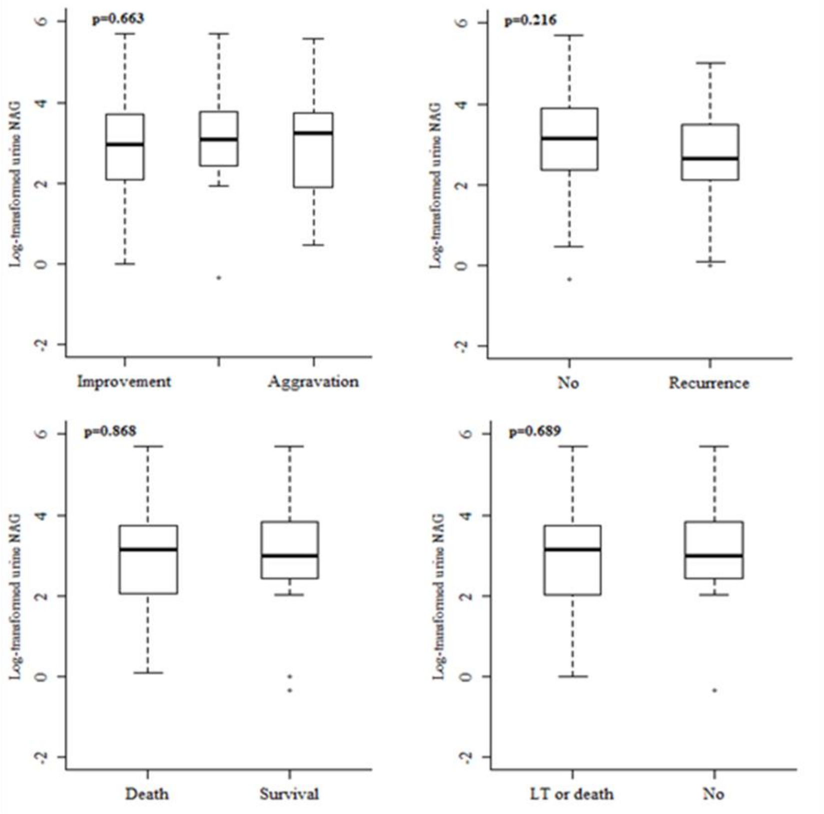

Supplementary Figure S5. Relationship between delta urine NAG and response to terlipressin

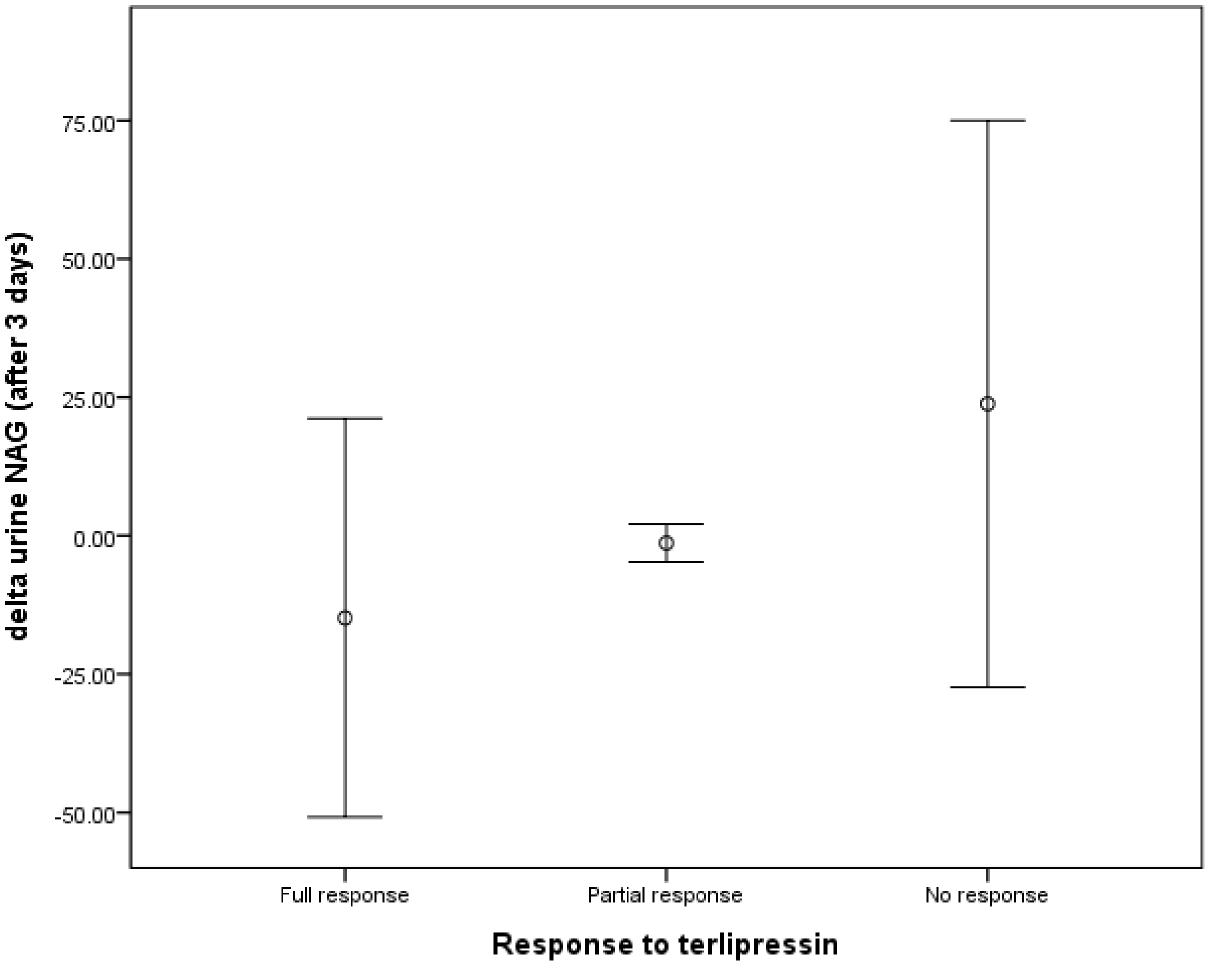

***P* = 0.383**
